# Insight into the Meat Quality Characteristics of Tibetan Sheep at Different Age Groups Using Metabolomics

**DOI:** 10.3390/ani16213362

**Published:** 2026-10-24

**Authors:** Gaoliang Bao, Hong Wei, Juane Bao

**Affiliations:** 1 College of Agriculture and Animal Husbandry, Qinghai University, Xining 810016, China; 2 Academy of Animal Science and Veterinary, Qinghai University, Xining 810016, China; 18146307169@163.com; 3 Gansu Key Laboratory of Herbivorous Animal Biotechnology, Faculty of Animal Science and Technology, Gansu Agricultural University, Lanzhou 730070, China; 18195616956@163.com

**Keywords:** Tibetan sheep, longissimus dorsi, fatty acid profile, volatile compounds, amino acids, intramuscular fat

## Abstract

Age-associated variation in Tibetan sheep meat quality and muscle metabolism remains incompletely understood. Eighteen clinically healthy intact male Tibetan sheep were divided into group by age: 6 (M6), 18 (M18) and 42 (M42) months, and their Longissimus dorsi muscles were evaluated for pH, colour, Warner–Bratzler shear force, cooking loss, proximate composition, amino acids, and fatty acids. Volatile compounds were profiled using headspace solid-phase microextraction coupled with gas chromatography–mass spectrometry, while muscle metabolites were assessed by untargeted liquid chromatography–mass spectrometry. The results showed that M42 sheep had the highest protein and fat content, as well as the greatest shear force. Notably, levels of monounsaturated and polyunsaturated fatty acids were the highest at M18, yielding distinct flavour profiles and indicating age-associated differences in fatty-acid composition rather than a defined metabolic transition point. Metabolomic analysis identified differential metabolites, primarily enriched in amino acid and lipid metabolism pathways. Correlation analysis revealed that key metabolites, including fumaric acid and linoleic acid, were significantly associated with meat quality parameters. This study elucidates the meat quality characteristics and metabolic basis of Tibetan sheep at different age groups, providing a theoretical foundation for improving meat quality.

## Simple Summary

Meat quality changes as animals grow, but the reasons behind these changes are not fully understood. This study investigated how the quality and nutritional characteristics of Tibetan sheep meat develop at different age groups. Sheep aged 6, 18 and 42 months were compared to evaluate changes in tenderness, nutrients, flavour-related compounds and natural substances produced during muscle development. The results showed that older sheep had higher protein and fat contents, while meat tenderness decreased with age. Sheep at 18 months showed notable advantages in the contents of flavour-related fatty acids, suggesting that this stage may represent an important period for balancing nutritional value and eating quality. Further analysis identified many naturally occurring substances in muscle that changed during growth, especially those related to protein and fat metabolism. Several key substances were closely associated with meat quality characteristics. These findings improve understanding of how Tibetan sheep meat quality develops and provide useful information for selecting appropriate production stages, improving meat processing, and supporting the sustainable development of Tibetan sheep farming.

## 1. Introduction

Tibetan sheep are a native ovine resource of the Qinghai–Tibet Plateau. After long-term adaptation to the low-temperature and hypoxic plateau environment, this breed has developed strong environmental resilience together with distinctive production traits [1,2]. For pastoral communities in this region, Tibetan sheep are of major economic importance because they provide high-quality mutton and contribute substantially to the sustainable development of local animal husbandry [3,4]. Tibetan mutton is particularly valued in premium markets owing to its high protein level, relatively low fat content and characteristic flavour [5]. Nevertheless, meat quality formation in Tibetan sheep is shaped by multiple interacting factors, including genetic background, production environment and developmental stage [6].

Among these factors, growth stage plays a pivotal role because it alters muscle physiology, metabolic activity and tissue architecture, thereby affecting tenderness, colour, water-holding capacity and flavour [7]. Increasing evidence indicates that the contents of amino acids, fatty acids and volatile compounds in livestock meat vary with age. In sheep, Wang et al. demonstrated clear age-related differences in both fatty acid composition and meat quality traits [8]. Chen et al. showed that linoleic acid and eicosapentaenoic acid were present at significantly higher levels in the muscle of 12-month-old Sunit sheep than in 18-month-old animals, and that both the polyunsaturated fatty acid (PUFA) content and the PUFA/saturated fatty acid (SFA) ratio exceeded those recorded at 24 months [9]. Using lipidomics, Tan et al. identified 201 age-responsive differential lipids in Chongming white goats, mainly involved in glycerophospholipid and glycerolipid metabolism [10]. In Tibetan sheep, Wang et al. used proteomic and phosphoproteomic approaches to show that glycolysis and the AMPK and HIF-1 signalling pathways are closely involved in the developmental regulation of muscle tenderness [11]. In addition, Ma et al. reported that variation in meat quality across age groups in Tibetan sheep was strongly associated with shifts in rumen microbiota and related gene expression [12]. As supplementary evidence from a monogastric species, Galli et al. found that, although the overall amino acid profile of growing-finishing pigs changed with age, the content of umami amino acids remained relatively stable [13]. Nevertheless, the metabolic mechanisms underlying age-dependent differences in Tibetan sheep meat quality remain insufficiently understood; conventional assessments do not characterise the accompanying small-molecule metabolic variation and integrating untargeted metabolomics with meat-quality traits and flavour precursors may identify candidate metabolites and pathways. Even so, the metabolic mechanisms underlying age-dependent differences in Tibetan sheep meat quality remain insufficiently understood. Although conventional meat-quality assessments and fatty acid analyses describe age-associated phenotypic and compositional differences, they do not provide a broad characterisation of the accompanying small-molecule metabolic variation. Integrating untargeted metabolomics with measurements of meat-quality traits and potential flavour precursors may therefore identify candidate metabolites and pathways associated with these differences.

As an important branch of systems biology, metabolomics provides comprehensive information on small-molecule metabolites and therefore reflects physiological state and metabolic perturbation more directly than many other omics approaches [14]. In meat science, metabolomics has been widely used to explain the biochemical basis of quality variation. Yu et al. identified inosine monophosphate, creatine, carnosine and conjugated linoleic acid in the longissimus dorsi muscle of Nellore cattle as metabolites closely associated with tenderness [15]. NMR-based metabolomics has also shown that energy-related compounds, especially creatine, affect pH and water-holding capacity in duck meat [16]. In Tibetan mutton, Ma et al. found through untargeted metabolomics that improved tenderness and water-holding capacity were associated with decreased adipocyte lipolysis and increased protein digestion and absorption pathways [17]. Liu et al. further showed that altitude-related variation in Tibetan mutton quality is linked to HIF-1α-mediated regulation of meat colour under hypoxic conditions [14]. Despite these advances, metabolomic studies specifically focused on growth-stage-dependent differences in Tibetan sheep meat quality remain scarce. Systematic identification of the key metabolites and metabolic pathways involved is therefore needed to better understand the biochemical basis of Tibetan mutton quality formation. Three age groups (6, 18, and 42 months) were selected to compare meat quality and muscle metabolic profiles among relatively young, young-adult, and mature-adult Tibetan sheep. Previous studies have reported age-associated variation in muscle quality and fatty acid composition in Tibetan sheep at 4 months, 1.5 years, 3.5 years, and 6 years of age [8,18]. Thus, the 18- and 42-month groups provide direct points of comparison with previous work, whereas the 6-month group represents younger animals. The present study was cross-sectional and did not follow individual animals over time. We hypothesised that Tibetan sheep from different age groups would differ in longissimus dorsi meat-quality traits, fatty acid composition, and volatile profiles, and that these differences would be accompanied by changes in small-molecule metabolites related to amino acid, lipid, and energy metabolism.

Accordingly, Tibetan sheep at three representative age groups, namely 6, 18 and 42 months, were included in this study. Physicochemical traits, proximate composition, amino acid and fatty acid profiles, volatile compounds and the untargeted muscle metabolome were evaluated within the same experimental framework. In contrast to previous studies that primarily addressed individual meat-quality traits, lipid composition or gene-expression patterns, the present study related growth-stage-associated variation in meat composition and volatile profiles to candidate small-molecule metabolites and metabolic pathways. The objectives were therefore to characterise compositional and metabolic differences among age groups and to identify candidate metabolite–quality associations relevant to tenderness, lipid composition and volatile profiles. The findings were expected to provide a chemical and metabolic basis for evaluating slaughter stage and Tibetan sheep meat quality under natural grazing conditions.

## 2. Materials and Methods

### 2.1. Animals and Sample Collection

Eighteen clinically healthy intact male Tibetan sheep were selected from the same free-grazing flock on an alpine pasture in Haiyan County, Qinghai Province, China, at approximately 3500 m above sea level. Six animals were included in each growth-stage group: 6 months (M6), 18 months (M18) and 42 months (M42). The pre-slaughter body weights were (24.52 ± 1.30) kg for M6, (34.53 ± 1.79) kg for M18 and (42.46 ± 2.04) kg for M42. Six independent biological replicates (one animal per replicate) were used per group, consistent with the replication commonly used in free-grazing sheep metabolomics. The number of clinically healthy intact males available in a single grazing flock constrained recruitment, and technical replicates were averaged to avoid pseudo-replication. Instead, animals with comparable health status and body condition were selected within each age group. All animals grazed the same pasture, received no concentrate supplementation and were slaughtered during the same sampling period. The contiguous natural alpine meadow pasture occupied by the experimental flock covered a total area of approximately 10.2 hectares (153 mu), with a stocking density of 1.76 sheep units per hectare. This stocking level complied with the theoretical carrying capacity standard (8.5 mu per sheep unit) issued for the grass–livestock balance zone of Haiyan County. All animals grazed the same pasture, received no concentrate supplementation and were slaughtered during the same sampling period. This design minimised short-term variation associated with pasture and slaughter season. Throughout the experimental period, all sheep were managed identically under a traditional free-grazing system and consumed only natural pasture herbage, composed mainly of species from the Gramineae, Salicaceae and Rosaceae family, but the nutritional composition of the pasture herbage was not determined in this study; nevertheless, all groups grazed on the same pasture and thus were exposed to a uniform nutritional plane. All experimental sheep had free access to natural mountain spring water distributed across the pasture. Mineral nutrient licks were evenly positioned in the grazing area for ad libitum consumption, providing supplemental macroelements (sodium, calcium, phosphorus) and essential trace elements (copper, zinc, selenium) without any supplementary feed. Slaughter was carried out in a commercial abattoir according to the National Standards of China [19]. All animal procedures complied with the Guidelines for the Breeding and Use of Experimental Animals issued by the Ministry of Science and Technology of the People’s Republic of China (Approval No. 2006-398). Animals were rested for 12 h after transportation to the abattoir; feed was withdrawn 24 h before slaughter, with free access to water until 2 h prior to slaughter. Head-only electrical stunning was applied, followed by exsanguination via severance of the carotid arteries and jugular veins, in strict accordance with the National Standards of China. After slaughter, longissimus dorsi muscle samples were collected within 30 min post-mortem, with an approximate weight of 20 g per sample. Longissimus dorsi (LD) muscle samples were excised from the left side of each carcass between the ninth and 13th ribs. Each sample was divided into three aliquots: One portion was maintained at 4 °C until 24 h post-mortem for measurement of ultimate pH (pH24) and instrumental colour; one was snap-frozen in liquid nitrogen and stored at −80 °C for metabolomic analysis; and the remaining portion was stored at −20 °C for the determination of cooking loss, tenderness, amino acids, fatty acids, protein, fat and moisture.

### 2.2. pH Determination

Ultimate muscle pH (pH24) was measured in the longissimus muscle at 24 h post-mortem using a portable penetration pH meter (Testo 205, Testo Instruments International Trading Ltd., Shanghai, China). Before measurement, the electrode was calibrated at the measurement temperature using pH 4.00 and 7.00 standard buffers. Three measurements were made at different positions in each sample, avoiding visible fat and connective tissue. The three readings were treated as technical replicates and averaged to obtain one pH24 value for each animal.

### 2.3. Colour Analysis

Colour measurements were made on freshly cut LD surfaces using a Minolta chromameter (CR-300, Minolta Camera Co., Osaka, Japan). Prior to use, the instrument was calibrated against a standard white tile under the following conditions: 8 mm aperture, D65 illuminant and 10° standard observer. Samples were allowed to bloom at 4 °C for 30 min before measurement. Three readings were recorded at different positions on each cross-section, and the results were expressed as L* (lightness), a* (redness) and b* (yellowness).

### 2.4. Tenderness Measurement

LD tenderness was assessed following the Wang method [20]. Longissimus dorsi muscle samples were cut into pieces approximately 2.5 cm thick and individually placed in plastic bags for water-bath cooking. Samples were sealed in plastic bags and heated in a 75 °C water bath until the internal temperature reached 70 °C, after which they were cooled to room temperature. Five cylindrical cores (1.27 cm diameter) were prepared from each sample parallel to the direction of the muscle fibres. Warner–Bratzler shear force (WBSF) was then measured perpendicular to the fibre orientation using a texture analyser (C-LM3B, Runhu Instrument Co., Ltd., Guangzhou, China).

### 2.5. Cooking Loss

For cooking loss determination, approximately 300 g of LD muscle was placed in a plastic bag and heated until the core temperature reached 70 °C. After cooling to room temperature, surface moisture was removed with absorbent paper and the sample was reweighed. Cooking loss was calculated from the percentage reduction in sample weight during cooking.

### 2.6. Moisture, Crude Protein and Crude Fat

Moisture, crude protein and crude fat were determined according to the official methods of the Association of Official Analytical Chemists [21]. Moisture was measured by oven drying at 105 °C (AOAC Method 934.01), crude protein was measured by the Kjeldahl method (AOAC Method 990.03), and crude fat was measured by Soxhlet extraction with petroleum ether (AOAC Method 920.39). All analyses were performed in triplicate.

### 2.7. Analysis of Volatile Compounds

Volatile flavour compounds were analysed by headspace solid-phase microextraction coupled with gas chromatography–mass spectrometry (HS-SPME/GC-MS) (8890-5977C, Agilent Technologies Inc., Santa Clara, CA, USA) as described by Lyu et al. [22], with minor modifications. Briefly, 10.00 g of a meat sample was placed in a 40 mL headspace vial, followed by the addition of 2 g NaCl and 5 μL of 2-octanol internal standard solution (88.01 μg/L). The vial was immediately sealed with a PTFE-silicone septum and equilibrated in a 60 °C water bath for 10 min. A preconditioned 50/30 μm DVB/CAR/PDMS fibre was then exposed to the headspace for 30 min and subsequently desorbed in the GC injector at 250 °C for 5 min. Chromatographic separation was achieved on an OV1701 capillary column (30 m × 0.25 mm internal diameter, 0.25 μm film thickness). The oven programme began at 35 °C and was held for 5 min, increased to 260 °C at 5 °C/min, and then held for 10 min. High-purity helium (≥99.999%) was used as the carrier gas at 1.0 mL/min. The injector temperature was set at 250 °C, and injections were made in splitless mode. The mass spectrometer operated in electron impact ionisation mode at 70 eV. The ion source, transfer line and quadrupole temperatures were 230, 260 and 150 °C, respectively. Mass spectra were recorded in full-scan mode over the range *m*/*z* 40–600.

### 2.8. Amino Acid Analysis

Amino acid composition was determined using an L-8900 automatic amino acid analyser (Hitachi High-Tech Corporation, Tokyo, Japan) as described by Ye et al. [23], with minor modifications. Approximately 1.0 g of homogenised meat sample was accurately weighed into a hydrolysis tube and mixed with 10 mL of 6 mol/L hydrochloric acid containing 0.1% phenol. The hydrolysis tube was flushed with nitrogen for 2–3 min to remove air, then immediately sealed and maintained at 110 °C for 24 h. After cooling to room temperature, the tube was opened, and the hydrolysate was filtered and evaporated to dryness under reduced pressure at 60 °C using a rotary evaporator. The residue was redissolved in sodium citrate loading buffer (pH 2.20) and diluted to a defined volume, then filtered through a 0.22 μm membrane before analysis. Amino acids were identified and quantified using a certified amino acid mixture standard suitable for protein hydrolysates, and multipoint external calibration curves were constructed for individual amino acids. Three analytical injections were performed for each animal sample as technical replicates, and their mean was used for statistical analysis.

### 2.9. Fatty Acid Analysis

Fatty acids were extracted and methylated as described by Nguyen et al. [24]. Fatty acid methyl esters were then analysed on an SRI 8610C gas chromatograph ((SRI Instruments, Torrance, CA, USA)) equipped with a flame ionisation detector and an HP-88 capillary column (100 m × 0.25 mm internal diameter, 0.2 μm film thickness). The oven temperature was programmed from 140 °C to 240 °C at 5 °C/min, followed by a 15 min hold. Injector and detector temperatures were set at 260 and 250 °C, respectively. High-purity hydrogen served as the carrier gas at a flow rate of 40 mL/min. Detector gases were supplied at 40 mL/min hydrogen and 450 mL/min air, while nitrogen was used as the make-up gas at 1.2 mL/min. Fatty acids were identified by comparing retention times with those of the Supelco 37 FAME Mix (47885-U, Supelco, Bellefonte, PA, USA). Quantification was performed with triglyceride C11:0 as the internal standard, and the results were expressed as g/100 g meat.

### 2.10. Untargeted Metabolomics

Metabolites were extracted as described by Liang et al., with modifications [25]. Thawed samples were mixed with precooled methanol/acetonitrile/water (2:2:1, *v*/*v*/*v*), vortexed thoroughly, ultrasonicated at 4 °C for 30 min, kept at −20 °C for 10 min and centrifuged at 14,000× *g* for 20 min at 4 °C. The resulting supernatant was vacuum-dried and redissolved in 100 μL acetonitrile/water (1:1, *v*/*v*). After vortexing and a second centrifugation at 14,000× *g* for 15 min at 4 °C, the final supernatant was used for analysis. Quality control (QC) samples were prepared by pooling equal aliquots of all extracts and were used for system conditioning and stability monitoring. Theoretically, QC samples are the same. But there will be systematic errors in the process of sample extraction, detection or analysis, which will lead to differences between QC samples. Principle component analysis (PCA) was performed by R language gmodels (version v2.18.1, R Foundation for Statistical Computing, Vienna, Austria). The more dense the distribution of QC is, the more reliable the results are. Chromatographic separation was performed on an Agilent 1290 Infinity II UHPLC system (Agilent Technologies Inc., Santa Clara, CA, USA) equipped with a HILIC column maintained at 25 °C. Mobile phase A consisted of water containing 25 mM ammonium acetate and 25 mM ammonium hydroxide, whereas mobile phase B was acetonitrile. The gradient programme was as follows: 0–0.5 min, 95% B; 0.5–7 min, 65% B; 7–8 min, 40% B; 8–9 min, 40% B; 9–9.1 min, 95% B; and 9.1–12 min, 95% B. The flow rate was 0.5 mL/min, the injection volume was 2 µL, and the autosampler temperature was 4 °C. Samples were analysed in random order with periodic QC injections. Mass spectrometric detection was performed on a Triple TOF 6600 system (SCIEX, Framingham, MA, USA) equipped with an ESI source and operated in both positive and negative ion modes. Source parameters were set as follows: Gas1, 60; Gas2, 60; CUR, 30; temperature, 600 °C; and ISVF, ±5500 V. The TOF MS scan range was *m*/*z* 60–1000 with an accumulation time of 0.20 s, and the product ion scan range was *m*/*z* 25–1000 with an accumulation time of 0.05 s. MS/MS spectra were acquired in information-dependent acquisition (IDA) high-sensitivity mode with DP ±60 V and CE 35 ± 15 eV. IDA settings excluded isotopes within 4 Da and selected 10 candidate ions per cycle. A variable importance in projection (VIP) score of (O)PLS model was applied to rank the metabolites that best distinguished between two groups. The threshold of VIP was set to 1. In addition, *t*-test was also used as a univariate analysis for screening differential metabolites. Those with a *p* value of *t*-test < 0.05 and VIP ≥ 1 were considered differential metabolites between two groups. Metabolites were mapped to KEGG metabolic pathways for annotation and enrichment analysis. Six biological replicates per group (one independent sample per animal) were included, and pooled quality control samples were used as technical replicates to monitor the stability of the analytical system.

### 2.11. Statistical Analysis

All phenotypic results are expressed as mean ± standard deviation (SEM), with *n* = 6 independent animals per group. Before one-way ANOVA, normality and homogeneity of variance were assessed using the Shapiro–Wilk and Levene’s tests, respectively. Data satisfying both assumptions were compared by one-way ANOVA followed by Tukey’s HSD post hoc test; data violating these assumptions were compared by the Kruskal–Wallis test with Dunn’s post hoc correction. Unless otherwise stated, *p* < 0.05 was considered statistically significant for conventional meat-quality and compositional analyses. For the untargeted metabolomics, metabolites were screened by variable importance in projection (VIP ≥ 1) from the (O)PLS model together with *t*-test *p* < 0.05, q-values were additionally computed and used for KEGG pathway enrichment rather than as a hard discovery cutoff. Statistical analyses were performed using SPSS 22.0 (SPSS Inc., Chicago, IL, USA) and R.

## 3. Results

### 3.1. Meat-Quality Traits at Different Age Groups

As shown in Table 1, growth stage significantly affected several physicochemical traits of Tibetan sheep meat. Values with different lowercase letters are significantly different (*p* < 0.05). Shear force increased progressively with age and reached the highest value in M42 (49.76 N; *p* < 0.05). Muscle pH showed the same upward tendency, although the difference between M18 and M42 was not significant. By contrast, M6 samples exhibited the highest L* and a* values and the greatest cooking loss among the three groups (*p* < 0.05). Moisture content was higher in M6 than in M42 (*p* < 0.05), whereas M6 and M18 did not differ significantly. Protein and fat displayed the opposite trend: both were lower in M6 than in M42 (*p* < 0.05), with no significant difference between M6 and M18.

### 3.2. Amino Acid Composition at Different Age Groups

The amino acid profiles of Tibetan sheep meat at the three age groups are presented in Table 2. Aspartic acid, glycine, alanine, tyrosine and phenylalanine were not significantly affected by age. In contrast, threonine, valine, isoleucine and leucine were all higher in M42 than in M6 (*p* < 0.05). Serine, glutamic acid, lysine, arginine and total amino acid content also increased significantly with age (*p* < 0.05), whereas cysteine decreased. Moreover, the totals of both essential and non-essential amino acids were the highest in M42 and were significantly different from those of M6 and M18 (*p* < 0.05). No significant difference in these totals was observed between M6 and M18.

### 3.3. Fatty Acid Profiles at Different Age Groups

Age-dependent variation was also evident in the fatty acid composition of Tibetan sheep meat (Table 3). Nineteen fatty acids were identified in total, including nine saturated fatty acids and ten unsaturated fatty acids. C6:0, C16:0, C17:1n7, C18:2n6c and C20:5n3 were significantly lower in M42 than in M6 and M18 (*p* < 0.05), whereas these components did not differ significantly in M6 and M18; numerically, C18:2n6c was the highest at M18. C14:0 (myristic acid), C17:0 (heptadecanoic acid), C24:0 (lignoceric acid) and total SFAs increased continuously with age (*p* < 0.05). In contrast, C14:1n5 (myristoleic acid) and C16:1n7 (palmitoleic acid) decreased with age. C18:0 (stearic acid) also increased, although the difference between M6 and M18 was not significant. A different pattern was observed for C18:1n9c (oleic acid), C22:6n3 (docosahexaenoic acid), C18:3n3 (α-linolenic acid), total MUFAs and total PUFAs, all of which increased from M6 to M18 and then decreased, with maximal values recorded at M18 (*p* < 0.05). No significant age effect was detected for C10:0 (capric acid), C12:0 (lauric acid), C15:0 (pentadecanoic acid), C18:1n9t (elaidic acid) or C20:4n6 (arachidonic acid).

### 3.4. Volatile Organic Compounds at Different Age Groups

**Figure 1:**
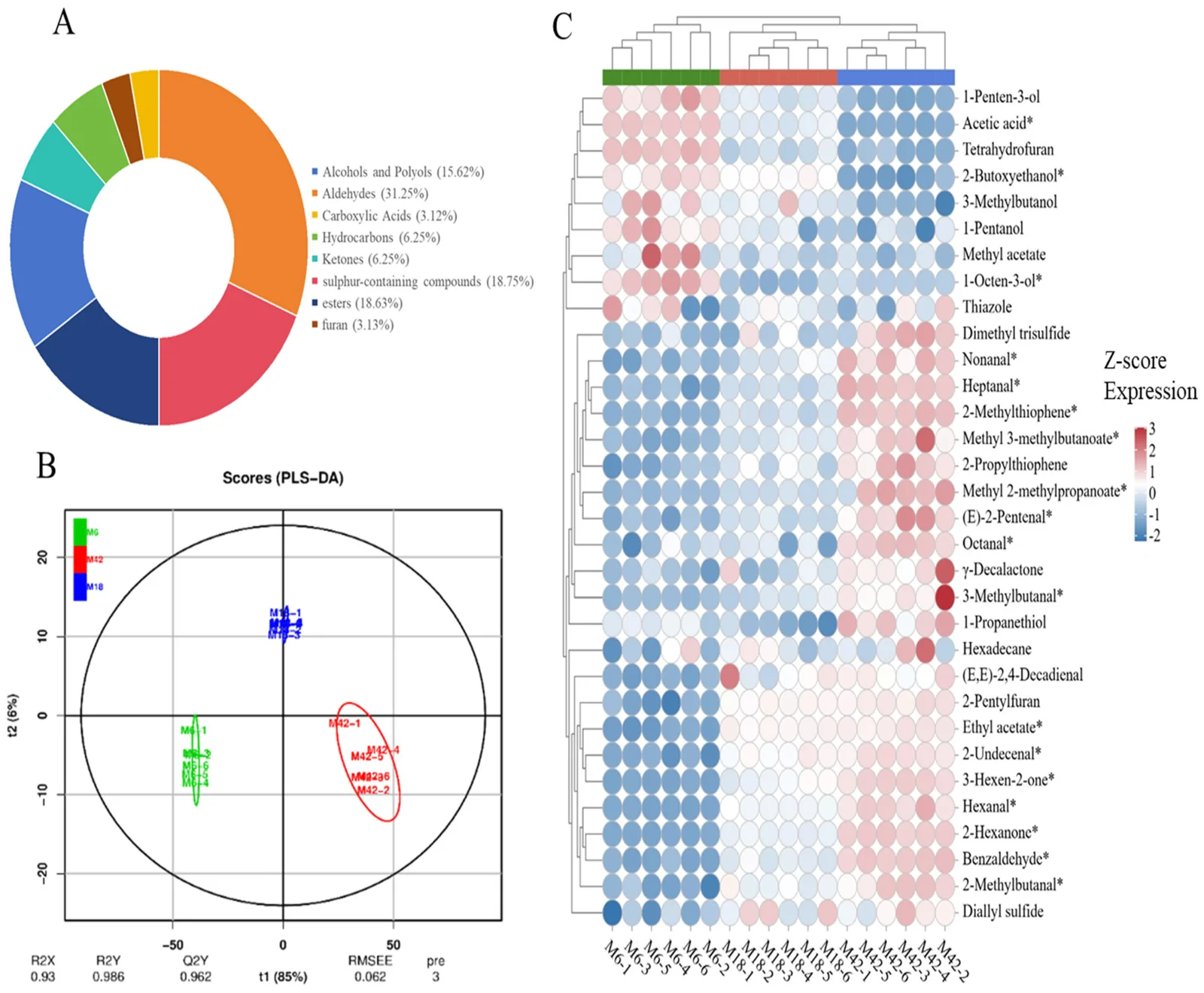
GC-MS-based profiling of volatile compounds in LD muscle from Tibetan sheep at different age groups. (**A**) Distribution of VOC classes. (**B**) PLS-DA score plot based on all detected VOCs. (**C**) Heatmap of VOC abundance; the horizontal axis indicates VOC clustering, and the colour scale represents normalised relative abundance (red, high; blue, low). An asterisk (*) indicates a significant difference (*p* < 0.05).

GC-MS profiling identified 32 volatile organic compounds (VOCs) in LD muscle across all groups (Figure 1), including 10 aldehydes, 5 alcohols, 2 ketones, 5 esters, 6 sulphur-containing compounds, 1 carboxylic acid, 2 hydrocarbons and 1 furan (Figure 1A). PC1 and PC2 explained 85.0% and 6.0% of the total variance, respectively, revealing clear separation among the three age groups in volatile-flavour composition (Figure 1B). Thirteen VOCs, namely (E)-2-pentenal, heptanal, hexanal, nonanal, 2-methylbutanal, 2-undecenal, 2-methylthiophene, methyl 2-methylpropanoate, methyl 3-methylbutanoate, 2-hexanone, 3-hexen-2-one, 3-methylbutanal and benzaldehyde, increased significantly with age (*p* < 0.05). In contrast, 2-butoxyethanol and acetic acid decreased progressively. Octanal and ethyl acetate also tended to increase, although octanal did not differ between M6 and M18 and ethyl acetate did not differ between M18 and M42. The abundance of 1-octanol showed the reverse pattern, decreasing with age and remaining similar between M18 and M42 (Figure 1C).

### 3.5. Metabolite Composition and Multivariate Statistical Analysis

**Figure 2:**
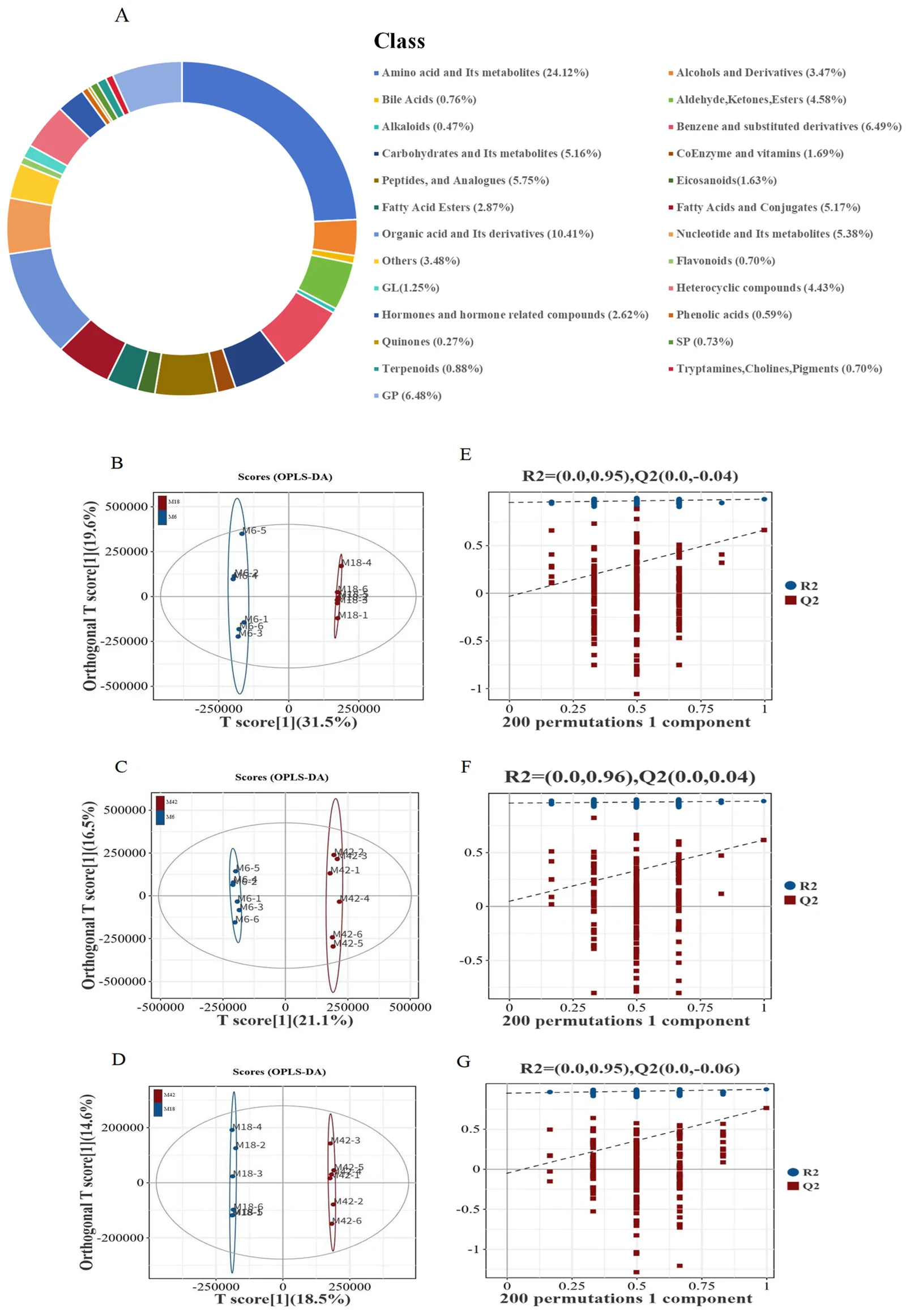
Multivariate analysis of the LD muscle metabolome. (**A**) Major metabolite classes and their proportions. (**B**–**D**) OPLS-DA score plots for M6 vs. M18, M6 vs. M42 and M18 vs. M42, respectively. (**E**–**G**) Permutation tests for the corresponding OPLS-DA models.

QC samples clustered tightly, indicating good repeatability and analytical stability of the metabolomic platform. In total, 1375 metabolites were detected in LD muscle and assigned to 25 major classes (Figure 2A). Amino acids and derivatives were the largest class (332 metabolites, 24.12%), followed by organic acids and derivatives (143, 10.41%), benzene derivatives (89, 6.49%) and glycerophospholipids (89, 6.48%). Thus, amino acid-related and organic acid-related compounds dominated the muscle metabolome. To compare the metabolic characteristics of M6, M18 and M42 sheep, pairwise OPLS-DA models were constructed (Figure 2B–D). Clear segregation of the groups was observed in all comparisons, indicating marked differences in their overall metabolic phenotypes. For the M6 vs. M18, M6 vs. M42 and M18 vs. M42 models, R^2^ > 0.9, while the corresponding Q^2^ intercepts were −0.04, 0.04 and −0.06, respectively, confirming high explanatory power without evident overfitting (Figure 2E–G). Among the three stages, M18 displayed the most compact within-group clustering and the clearest separation from the other groups.

### 3.6. Differential Metabolite Analysis

**Figure 3:**
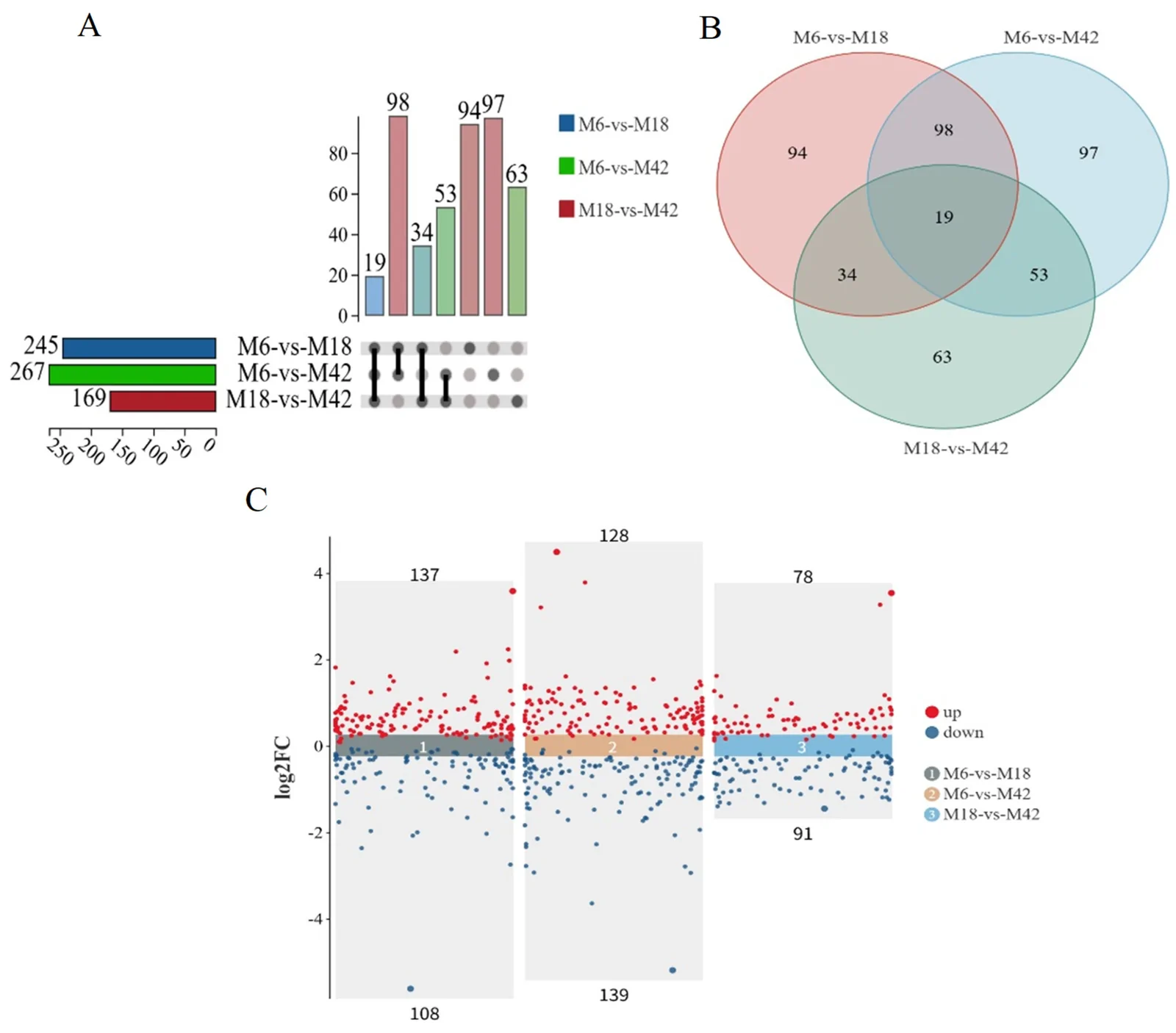
Differential metabolite comparison among M6, M18 and M42 LD muscle samples. (**A**,**B**) Venn diagrams showing shared and unique metabolites among the three pairwise comparisons. (**C**) Volcano plots of M6 vs. M18, M6 vs. M42 and M18 vs. M42. The vertical axis represents the logarithm of fold change in metabolite abundance between two groups.

A total of 681 differential metabolites were detected across the three pairwise comparisons. Their shared and specific distributions are illustrated in the Venn diagrams in Figure 3A,B, where 98, 34 and 53 overlapping metabolites were associated with the M6, M18 and M42 groups, respectively. The volcano plots in Figure 3C further show the direction and magnitude of metabolite changes. In the M6 vs. M18 comparison, 137 metabolites were up-regulated and 108 were down-regulated. In M6 vs. M42 comparison, 128 metabolites increased and 139 decreased. In M18 vs. M42 comparison, 78 metabolites were up-regulated and 91 were down-regulated. In all three comparisons, fatty acyls, amino acids and related metabolites represented the dominant differential classes.

### 3.7. KEGG Enrichment Analysis of Core Differential Metabolites

**Figure 4:**
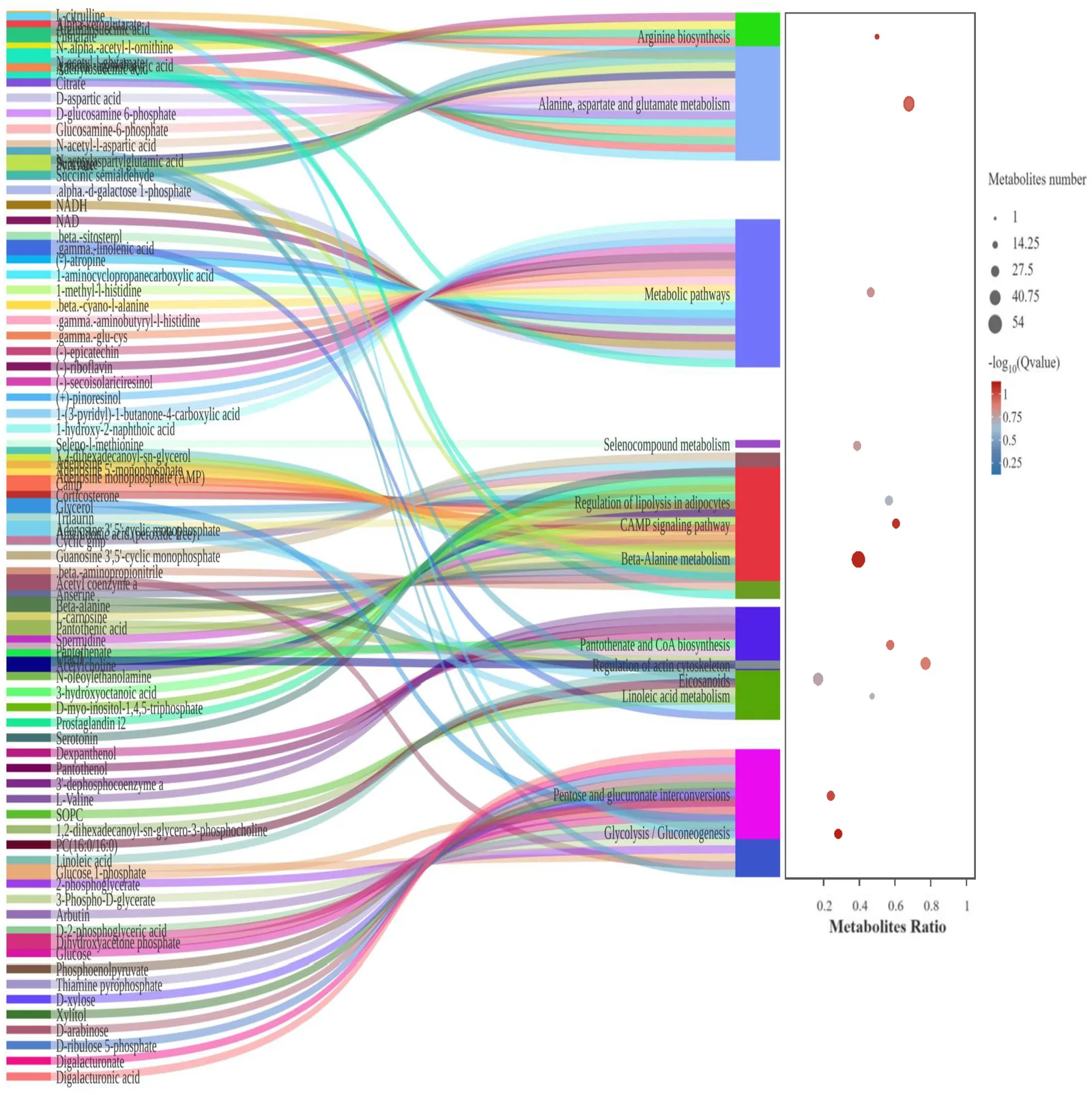
Sankey diagram of KEGG pathway enrichment for differential metabolites in Tibetan sheep at different age groups. The nodes on the left represent metabolites enriched in the corresponding pathways, and Metabolite Ratio indicates the proportion of differential metabolites assigned to each pathway.

When the screening criteria were tightened to *p* < 0.05 and VIP > 1.5, 114 core differential metabolites were retained for pathway analysis. KEGG enrichment using MetaboAnalyst 5.0 showed that these metabolites were mainly involved in arginine biosynthesis; alanine, aspartate and glutamate metabolism; global metabolic pathways; regulation of lipolysis in adipocytes; and the cAMP signalling pathway (Figure 4). Arginine biosynthesis and alanine, aspartate and glutamate metabolism are closely related to nitrogen homeostasis and carbon-nitrogen allocation, and may therefore influence carnosine formation. The cAMP signalling pathway participates in the regulation of adipocyte lipolysis. Linoleic acid metabolism also emerged as a pathway of interest because of its likely contribution to lipid homeostasis within the broader metabolic network.

### 3.8. Correlation Analysis Between Meat Quality and Major Differential Metabolites

**Figure 5:**
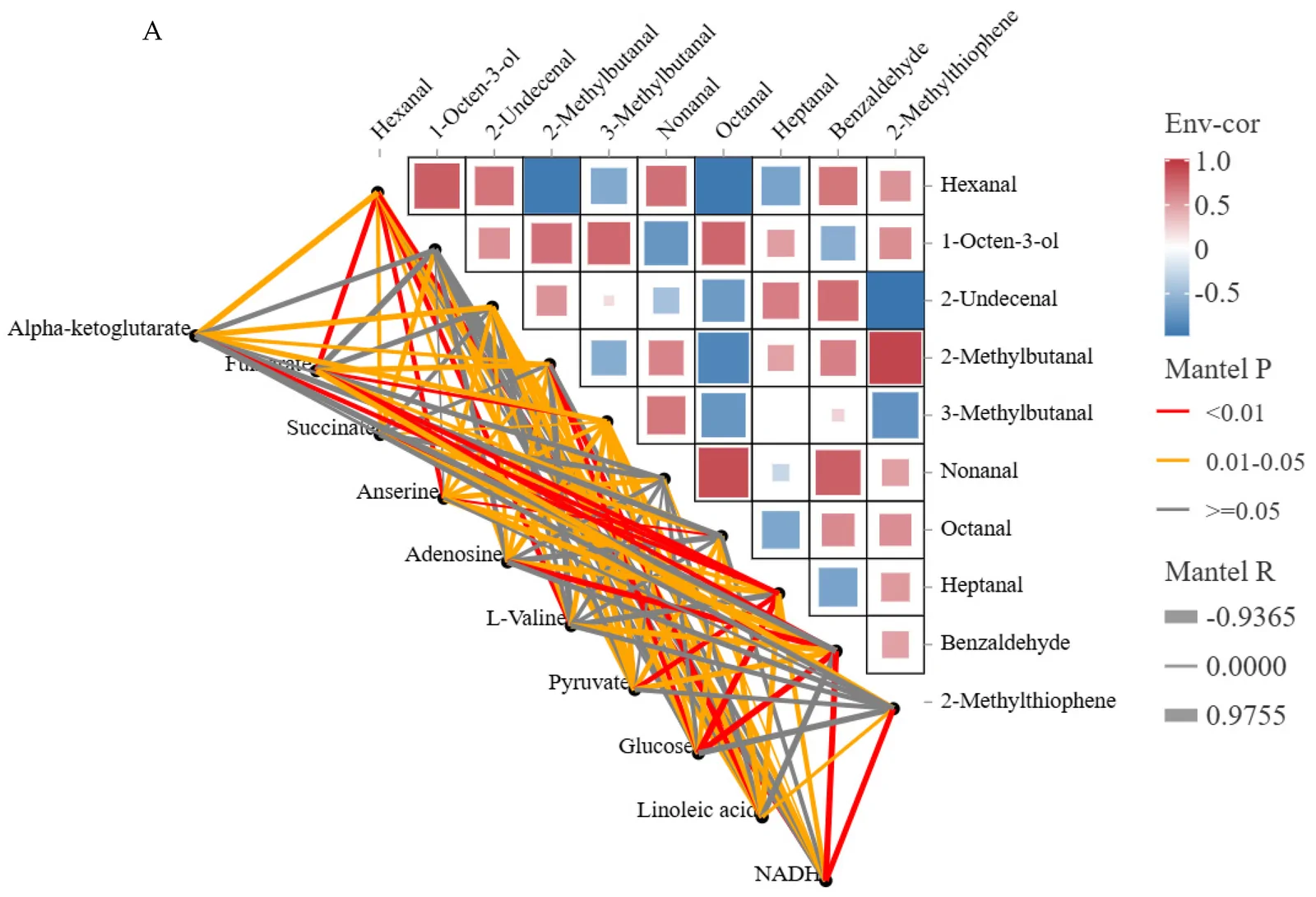

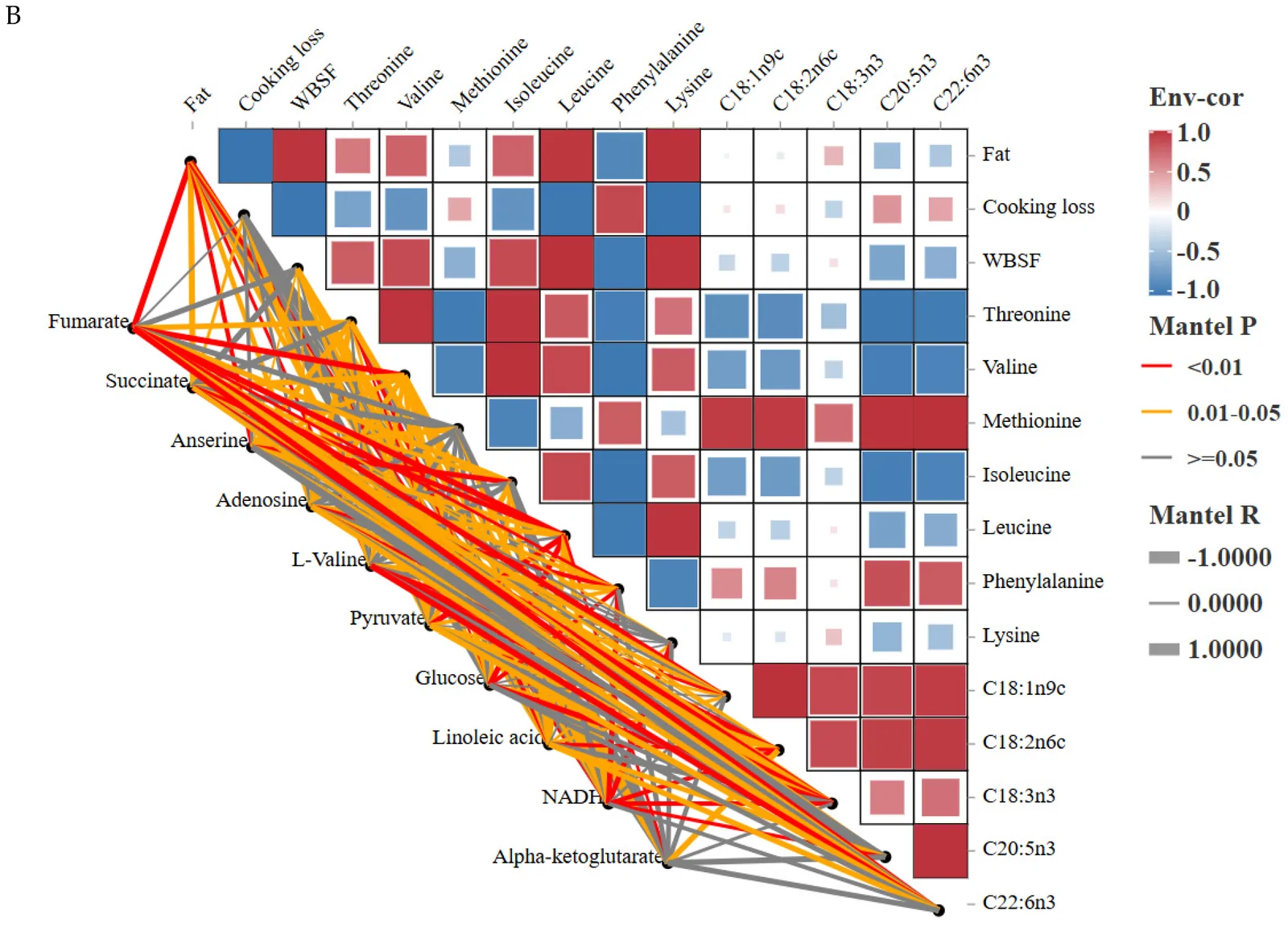
Mantel correlation analysis between phenotypic traits and major differential metabolites. (**A**) Correlations between metabolites and flavour compounds. (**B**) Correlations between metabolites and meat-quality traits. The colour gradient (blue to red) indicates the Pearson correlation coefficient (red, positive; blue, negative), indicating pairwise correlations between individual metabolites and individual traits. Line width is proportional to the Mantel correlation coefficient for significant relationships (*p* < 0.05), indicating significant relationships between metabolite sets and phenotypic trait groups.

To explore the relationship between metabolic variation and meat phenotype, a Mantel heatmap was generated using the ten most significant differential metabolites (Figure 5). Several metabolites, including fumarate, succinate, anserine, L-valine, glucose, linoleic acid and alpha-ketoglutarate, showed significant correlations with meat quality and nutritional indices such as fat content, cooking loss, Warner–Bratzler shear force (WBSF), leucine, phenylalanine, lysine, C20:5n3 and C22:6n3. Significant associations were also observed between fumarate, succinate, anserine, adenosine, L-valine, pyruvate, linoleic acid and alpha-ketoglutarate and representative volatile compounds, including 1-octanol, 2-methylbutanal, octanal and heptanal. Particularly strong correlations (*p* < 0.01) were detected between fat content, WBSF, lysine, C20:5n3 and C22:6n3 and the metabolites fumarate, succinate, adenosine, L-valine and linoleic acid.

## 4. Discussion

Tibetan sheep have evolved under the harsh conditions of the Qinghai–Tibet Plateau, and their meat quality is shaped during growth by coordinated adjustments in protein deposition, lipid turnover, energy utilisation and redox balance [26]. Although multi-omics approaches have gradually improved our understanding of developmental variation in Tibetan sheep muscle, the contribution of age-related metabolites to final meat quality has not been comprehensively defined. By integrating phenotypic measurements with untargeted metabolomics, the present study provides a clearer view of how growth stage influences both the nutritional and sensory attributes of Tibetan mutton.

The progressive rise in shear force from M6 to M42 indicates a decline in tenderness with age. A plausible explanation is the increasing density and cross-linking of intramuscular collagen as connective tissue matures. Meanwhile, younger animals retained more moisture and less fat, which is consistent with their still-developing muscle and adipose tissue. Similar patterns have been reported at the transcriptomic level, where shear force increased with age but intramuscular fat peaked at 1.5 years in Tibetan sheep [18]. This supports the view that mid-age animals may offer a practical balance between tenderness and the accumulation of flavour precursors. The higher L* and a* values observed in M6 meat also indicate that age influences colour development. Because myoglobin concentration and redox state are major determinants of meat colour [27], the lighter appearance of younger meat is likely related to lower myoglobin content and reduced mitochondrial density in muscle fibres. In plateau animals, hypoxia can additionally affect colour formation by activating HIF-1α signalling, promoting oxidative stress and disturbing glutathione redox homeostasis, which in turn compromises myoglobin stability [14]. The greater cooking loss in M6 further suggests weaker water-holding capacity. This trait is strongly influenced by post-mortem pH decline, protein denaturation and the extent of contraction of the myofibrillar lattice [28]. Differences in muscle energy metabolism under distinct feeding systems have also been shown to influence Tibetan sheep meat quality [29]. In terms of nutrient composition, the increase in protein and fat, together with the decrease in moisture, reflects normal muscle maturation, during which protein deposition gradually becomes dominant and adipose tissue develops further [30]. Likewise, Chen et al. reported that the muscle fatty acid profile of 18-month-old sheep was more advantageous than that of older animals, suggesting superior meat-quality potential at the young-adult stage [31].

The amino acid data provide further evidence that muscle nutritional quality changes as Tibetan sheep age. Higher concentrations of threonine, valine, isoleucine, leucine, serine, glutamic acid, lysine, arginine and total amino acids in M42 meat point to greater protein deposition and altered amino acid demand in mature muscle. Branched-chain amino acids are especially relevant because, beyond serving as substrates for protein synthesis, they function as metabolic signals that activate the mTOR pathway and regulate protein turnover [32]. In contrast, aspartic acid, glycine, alanine, tyrosine and phenylalanine remained relatively stable across stages. This agrees with the observation of Jung et al. [33], who found no significant age-related variation in umami amino acids in Hanwoo beef, suggesting that these compounds may be under tighter homeostatic control or less sensitive to developmental stage. The progressive decline in cysteine is particularly noteworthy. As a precursor of glutathione, cysteine supports the intracellular antioxidant system, and its depletion in older animals may therefore indicate greater oxidative demand. This interpretation is consistent with previous evidence that high-altitude oxidative stress increases the utilisation of antioxidant defences in Tibetan sheep [14].

Age-related remodelling of lipid metabolism was clearly reflected in the fatty acid profile. C6:0, C16:0, C17:1n7, C18:2n6c and C20:5n3 were lower in M42 than in M6 and M18, indicating that these components did not accumulate continuously with age; this pattern differs from the progressive age-related rise in C14:0, C17:0, C18:0, C24:0 and total SFA observed in the same dataset. Comparable age effects on muscle fatty acids have been described in Tibetan sheep, and the 1.5-year-old group in that work showed the most desirable overall profile across different muscles [8]. In the present study, C18:1n9c, C22:6n3, C18:3n3, total MUFAs and total PUFAs all peaked at M18, reinforcing the idea that this stage is metabolically advantageous for lipid deposition and flavour development. From a nutritional perspective, C18:2n6c is noteworthy because it is an essential fatty acid for humans. It also contributes to aroma generation, as oxidation and esterification products derived from this fatty acid can form volatile aldehydes and related flavour compounds [34]. The concomitant decline in C14:1n5 and C16:1n7 and increase in C18:0 may reflect developmental changes in desaturation capacity. Stearoyl-CoA desaturase is the principal enzyme that converts saturated fatty acids into monounsaturated fatty acids, and its activity is known to respond to hormonal and nutritional regulation [35]. In agreement with our results, Wang et al. found that hindleg stearic acid content was significantly higher in 6.0-year-old sheep than in 1.5-year-old sheep [8].

Flavour differences among age groups were also evident at the volatile level. The 32 VOCs identified here were dominated by aldehydes, alcohols, ketones, esters and sulphur-containing compounds, and multivariate analysis clearly separated the three age groups. A GC-IMS study of four Chinese indigenous sheep breeds likewise found aldehydes, alcohols and ketones to be the predominant volatile classes, with relatively high signals for nonanal, octanal, heptanal, 3-methylbutanal and hexanal [36]. In our data, thirteen compounds, including (E)-2-pentenal, heptanal, hexanal, nonanal and 2-methylbutanal, increased with age. This is chemically plausible because hexanal, heptanal and nonanal are classic oxidation products of linoleic acid and arachidonic acid [37], whereas 2-methylbutanal and 3-methylbutanal arise from the Strecker degradation of branched-chain amino acids [38]. Another GC-IMS study identified 66 volatile flavour compounds and showed that 2-month-old yak muscle contained more alcohols, terpenes and esters, whereas 6- and 12-month-old yak muscle contained higher levels of ketones and aldehydes [39]. Heptanal, hexanal and nonanal have also been identified as major characteristic aroma compounds in Black Tibetan sheep at different ages using OPLS-DA and relative odour activity value analysis. By comparison, 1-octanol declined with age in the present study. This alcohol is thought to arise mainly from arachidonic acid oxidation and is associated with mushroom-like, grassy and vegetable-like notes [40]. It has also been recognised as an important flavour compound in sheep meat [41].

Untargeted metabolomics further clarified the biochemical basis of these phenotypic differences. Amino acid derivatives and organic acid derivatives accounted for the largest proportion of the LD metabolome, in line with the metabolomic comparison of stall-fed and grazing Tibetan sheep reported by Wang et al. [29]. The clear separation of the three age groups in OPLS-DA space, together with the satisfactory model statistics, indicates that growth stage profoundly reshapes muscle metabolic status. Notably, M18 displayed the tightest internal clustering and the clearest discrimination compared to the other two age stages. Although M18 exhibited a distinct metabolic profile, the cross-sectional design does not establish 18 months as a metabolic transition point. Moreover, correlations between metabolites and meat-quality traits suggest potential biological associations but do not demonstrate direct causal relationships. This inference is consistent with the data on Huai goat researched by Han et al. [42], who reported advantages in intramuscular fat and related metabolic indices in 1.5-year-old Tibetan sheep. Across all comparisons, fatty acyls, amino acids and their related metabolites dominated the differential metabolite set. KEGG enrichment pointed mainly to arginine biosynthesis; alanine, aspartate and glutamate metabolism; global metabolic pathways; regulation of lipolysis in adipocytes; and cAMP signalling. These pathways are intimately connected with nitrogen balance, carbon–nitrogen flux distribution, lipid mobilisation and energy regulation, and therefore provide a plausible biochemical framework for the observed quality differences. Carnosine production may be especially relevant in this context, as Li et al. showed that this dipeptide contributes to antioxidant capacity and pH buffering in muscle and is closely linked to meat quality [43].

The correlation analysis highlighted several candidate metabolites that may connect metabolic regulation with phenotypic outcome. Fumarate and succinate are intermediates of the tricarboxylic acid cycle and were associated with several meat-quality traits in the present study. These associations may reflect differences in muscle energy metabolism. However, mitochondrial function, oxidative stress, and collagen cross-linking were not directly measured. Therefore, the underlying mechanisms remain to be established. Comparable associations between differential metabolites and phenotypic traits, including shear force, cooking loss, elasticity and flavour, have been reported previously in Tibetan sheep muscle [14]. L-valine, a branched-chain amino acid, was also significantly related to shear force, which may reflect its role in regulating muscle protein metabolism. This is compatible with the evidence that branched-chain amino acids promote protein synthesis via mTOR signalling while suppressing autophagic protein degradation [44]. Likewise, the relationship between linoleic acid and both fat content and PUFA abundance is expected, given its dual function as a membrane phospholipid component and a precursor of longer-chain polyunsaturated fatty acids. The negative association between anserine and 1-octanol is also notable. Because anserine has recognised antioxidant and metal-chelating properties [45], lower 1-octanol levels in samples with higher anserine may reflect reduced lipid oxidation. Collectively, these results indicate that age-dependent changes in Tibetan sheep meat quality arise from interacting shifts in protein metabolism, lipid metabolism, energy flux and redox homeostasis. This study was limited by the relatively small sample size and cross-sectional design. Furthermore, body weight and physiological maturity may have influenced meat quality and muscle metabolite profiles. Consequently, their effects cannot be fully distinguished from age-associated differences.

## 5. Conclusions

This study systematically compared growth-stage-related differences in meat quality and muscle metabolite composition in Tibetan sheep. Meat from M42 animals contained the highest protein and fat contents and showed the greatest shear force, whereas MUFAs and PUFAs were most abundant in M18 animals, giving this age stage a distinct volatile profile. Differential metabolites were mainly enriched in amino acid- and lipid-related pathways. Among them, fumaric acid and linoleic acid were strongly associated with meat-quality traits. These findings provide a biochemical basis for selecting an appropriate slaughter age and for improving Tibetan mutton quality.

## Figures and Tables

**Table 1:** Meat-quality traits of Tibetan sheep at different age groups.

Index	M6	M18	M42	*p*-Values
WBSF (N)	30.12 ± 2.05 ^c^	43.14 ± 3.27 ^b^	49.76 ± 3.58 ^a^	<0.001
pH (24 h)	5.42 ± 0.46 ^b^	5.73 ± 0.39 ^a^	5.84 ± 0.57 ^a^	0.004
L*	39.98 ± 1.32 ^a^	37.05 ± 2.87 ^b^	36.01 ± 2.55 ^bc^	0.015
a*	19.04 ± 1.02 ^a^	18.43 ± 0.83 ^b^	17.95 ± 0.90 ^b^	0.027
b*	9.26 ± 0.87	8.98 ± 0.66	9.05 ± 0.84	0.757
Cooking loss (%)	35.15 ± 1.58 ^a^	31.26 ± 1.04 ^b^	30.43 ± 1.67 ^bc^	0.003
Moisture (%)	75.12 ± 3.81 ^a^	74.49 ± 4.15 ^ab^	71.08 ± 3.25 ^b^	<0.001
Protein (%)	20.03 ± 1.29 ^b^	21.78 ± 1.42 ^ab^	22.08 ± 1.57 ^a^	0.014
Fat (%)	1.68 ± 0.07 ^b^	2.53 ± 0.08 ^ab^	2.67 ± 0.05 ^a^	0.003

Lower case letters represent significant differences in indicators among Tibetan sheep at different age groups (*p* < 0.05). The same applies to the following tables.

**Table 2:** Amino acid composition and content in longissimus dorsi muscle of Tibetan sheep at different age groups.

Amino Acids (g/100 g)	M6	M18	M42	*p*-Values
Aspartic acid	1.35 ± 0.07	1.47 ± 0.08	1.52 ± 0.09	0.062
Threonine ^1^	1.41 ± 0.02 ^bc^	1.47 ± 0.09 ^b^	2.38 ± 0.07 ^a^	0.018
Serine	0.65 ± 0.01 ^c^	0.83 ± 0.01 ^b^	0.99 ± 0.02 ^a^	0.003
Glutamic acid	2.45 ± 0.05 ^c^	2.82 ± 0.07 ^b^	3.56 ± 0.10 ^a^	<0.001
Glycine	0.98 ± 0.02	1.04 ± 0.02	1.10 ± 0.01	0.371
Alanine	2.09 ± 0.17	1.97 ± 0.09	2.10 ± 0.07	0.159
Cysteine	0.34 ± 0.01 ^a^	0.31 ± 0.01 ^b^	0.22± 0.01 ^c^	0.007
Valine ^1^	1.21 ± 0.08 ^b^	1.33 ± 0.06 ^b^	1.75 ± 0.03 ^a^	0.042
Methionine ^1^	0.34 ± 0.06	0.35 ± 0.08	0.31 ± 0.07	0.813
Isoleucine ^1^	0.97 ± 0.07 ^b^	1.06 ± 0.08 ^b^	1.37 ± 0.06 ^a^	0.047
Leucine ^1^	1.81 ± 0.07 ^b^	1.97 ± 0.05 ^ab^	2.06 ± 0.08 ^a^	0.016
Tyrosine	1.72 ± 0.10	1.65 ± 0.08	1.73 ± 0.07	0.425
Phenylalanine ^1^	1.23 ± 0.07	1.13 ± 0.06	0.98 ± 0.08	0.073
Lysine ^1^	1.89 ± 0.05 ^c^	2.410 ± 0.03 ^b^	2.54 ± 0.04 ^a^	<0.001
Histidine	1.07 ± 0.05 ^b^	1.60 ± 0.02 ^a^	1.58 ± 0.06 ^a^	0.008
Arginine	0.91 ± 0.04 ^c^	1.62 ± 0.05 ^b^	2.05 ± 0.08 ^a^	<0.001
Proline	0.87 ± 0.03 ^bc^	0.93± 0.04 ^b^	1.64 ± 0.03 ^a^	0.012
EAA	8.86 ± 0.76 ^bc^	9.72 ± 0.82 ^b^	11.39 ± 0.68 ^a^	0.025
NEAA	12.43 ± 1.01 ^bc^	14.24 ± 1.05 ^b^	16.49 ± 1.06 ^a^	0.014
TAA	21.29 ± 1.32 ^c^	23.96 ± 1.23 ^b^	27.88 ± 1.43 ^a^	<0.001

The superscript “1” denotes essential amino acids. Within the same parameter, means bearing different lowercase letters differ significantly (*p* < 0.05).

**Table 3:** Fatty acid profiles in longissimus dorsi muscle of Tibetan sheep at different age groups.

Fatty Acids (g/100 g)	M6	M18	M42	*p*-Values
C6:0	0.53 ± 0.02 ^a^	0.55 ± 0.03 ^a^	0.32 ± 0.02 ^b^	0.031
C10:0	0.76 ± 0.06	0.68 ± 0.07	0.78 ± 0.06	0.384
C12:0	0.95 ± 0.08	0.93 ± 0.08	1.06 ± 0.09	0.670
C14:0	1.31 ± 0.17 ^c^	2.15 ± 0.15 ^b^	2.87 ± 0.26 ^a^	0.001
C14:1n5	0.89 ± 0.02 ^a^	0.65 ± 0.04 ^b^	0.53 ± 0.03 ^c^	0.007
C15:0	0.81 ± 0.04	0.83 ± 0.03	0.82 ± 0.05	0.170
C16:0	14.31 ± 1.23 ^a^	14.42 ± 1.03 ^a^	10.75 ± 0.96 ^b^	0.025
C16:1n7	1.81 ± 0.09 ^a^	1.59 ± 0.11 ^b^	0.92 ± 0.07 ^c^	0.016
C17:0	0.83 ± 0.06 ^c^	1.01 ± 0.08 ^b^	1.77 ± 0.15 ^a^	0.003
C17:1n7	1.05 ± 0.07 ^a^	1.18 ± 0.09 ^a^	0.65 ± 0.03 ^b^	0.038
C18:0	15.87 ± 1.46 ^b^	16.02 ± 1.91 ^b^	24.41 ± 2.47 ^a^	0.042
C18:1n9t	3.25 ± 0.08	3.11 ± 0.09	3.19 ± 0.10	0.739
C18:1n9c	32.10 ± 1.04 ^b^	34.98 ± 1.21 ^a^	29.53 ± 1.16 ^c^	0.006
C18:2n6c	5.46 ± 0.48 ^a^	6.53 ± 0.79 ^a^	4.37 ± 0.53 ^b^	0.010
C18:3n3	1.24 ± 0.06 ^b^	3.95 ± 0.11 ^a^	1.15 ± 0.05 ^b^	0.004
C20:4n6	2.97 ± 0.12	3.12 ± 0.10	2.96 ± 0.09	0.843
C20:5n3	1.57 ± 0.05 ^a^	1.63 ± 0.04 ^a^	1.23 ± 0.04 ^b^	0.004
C22:6n3	0.79 ± 0.02 ^b^	0.87 ± 0.05 ^a^	0.53 ± 0.03 ^c^	0.015
C24:0	1.15 ± 0.07 ^c^	2.64 ± 0.09 ^b^	3.47 ± 0.15 ^a^	0.003
SFA	36.52 ± 1.14 ^c^	39.23 ± 1.35 ^b^	46.25 ± 2.27 ^a^	0.005
MUFA	39.10 ± 1.12 ^b^	41.51 ± 1.27 ^a^	34.82 ± 2.10 ^c^	0.027
PUFA	12.03 ± 0.88 ^b^	16.10 ± 1.05 ^a^	10.24 ± 0.89 ^c^	0.036

SFA, saturated fatty acids; MUFA, monounsaturated fatty acids; PUFA, polyunsaturated fatty acids. Within the same parameter, means bearing different lowercase letters differ significantly (*p* < 0.05).

## Data Availability

The data supporting the findings of this study are available from the corresponding author upon reasonable request.
